# Cu_2_Sn_1−*x*_Gd_*x*_S_3_ thin films for photocatalytic degradation of methylene blue

**DOI:** 10.1039/d5na00655d

**Published:** 2025-08-26

**Authors:** Serap Yiğit Gezgin, Şilan Baturay, M. Zafer Köylü, Mohamed A. Basyooni-M. Kabatas, Hamdi Şükür Kiliç

**Affiliations:** a Department of Physics, Faculty of Science, University of Selçuk 42031 Selcuklu Konya Turkey; b Department of Physics, Faculty of Science, Dicle University 21280 Diyarbakir Turkey; c Micro and Nano Engineering Group, Department of Precision and Microsystems Engineering, Delft University of Technology Mekelweg 2 2628 CD Delft The Netherlands m.kabatas@tudelft.nl m.a.basyooni@gmail.com; d Department of Nanotechnology and Advanced Materials, Graduate School of Applied and Natural Science, Selçuk University 42030 Konya Turkey; e Solar Research Laboratory, Solar and Space Research Department, National Research Institute of Astronomy and Geophysics 11421 Cairo Egypt; f Department of Metallurgical and Materials Engineering, Faculty of Engineering, University of Dokuz Eylül İzmir Turkey

## Abstract

Thin films of Cu_2_Sn_1−*x*_Gd_*x*_S_3_ were prepared on soda-lime glass substrates using spin coating in a sulfur-rich environment. We investigated how doping Cu_2_SnS_3_ with gadolinium (Gd) affected its structural, morphological, and optical properties using X-ray diffraction (XRD), Raman spectroscopy, field emission scanning electron microscopy (FE-SEM), and UV-Vis spectroscopy. XRD showed that all samples had a polycrystalline monoclinic structure, while FE-SEM revealed a mix of spherical and polygon-shaped grains. Optical analysis indicated an energy gap ranging from 2.10 to 1.50 eV, increasing with higher Gd content. The films exhibited increasing transmittance with longer wavelengths in the UV-Vis region. When tested for photocatalytic activity, the Cu_2_Sn_1−*x*_Gd_*x*_S_3_ films effectively degraded methylene blue (MB) dye under visible light within 220 minutes. The Cu_2_Sn_0.25_Gd_0.75_S_3_ film showed the highest degradation efficiency (90.77%) with a rate constant (*k*) of 0.093 min^−1^. Adjusting the pH of the dye solution improved the performance, reaching 90.77% degradation efficiency at pH 10, compared to 41.25% and 61.94% at pH 4 and 7, respectively. Tests with scavengers EDTA-Na, IPA, and BQ resulted in degradation efficiencies of 61.78%, 78.24%, and 43.56%, respectively, highlighting that the highest efficiency (90.77%) occurred without scavengers. The results show promising potential for these films in treating pollutants in industrial and domestic wastewater systems.

## Abstract 2

Cu_2_Sn_1−*x*_Gd_*x*_S_3_ thin films exhibited enhanced structural and optical properties, achieving 90.77% degradation of methylene blue under visible light, demonstrating strong potential for wastewater treatment.

## Graphical abstract

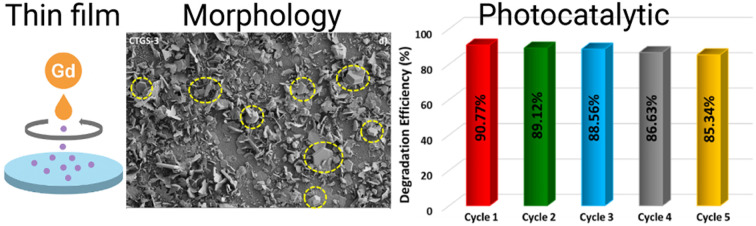

## 1. Introduction

There is a growing demand for energy and a faster reduction in non-renewable energy sources, making it imperative to find non-renewable energy sources. One of the most prominent non-renewable energy sources is the solar cell. Due to the high absorbance of thin-film solar cells and improved photovoltaic (PV) performance, thin-film solar cells are receiving considerable attention worldwide. Materials such as CuIn(Se, S)_2_, CuInGaSe_2_, and CdTe are used as solar absorbing materials to fabricate thin film solar cells. CuInGaSe_2_ (CIGS) solar cells with a Cd-free structure achieved a record 23.64% efficiency.^1^ This was obtained *via* applying a ‘hockey stick’-shaped GGI profile, where the gallium (Ga) content in solution remained constant in the upper half of the absorber while maintaining a high concentration level close to the back contact, significantly reducing open-circuit voltage (*V*_OC_) losses. However, the presence of poisonous elements and the high deposition costs of CuInGaSe_2_ solar cells have driven the examination of alternative materials. Among them, Cu_2_ZnSnS_4_ (CZTS) stands out as a promising photocatalytic and solar cell absorber material, offering a viable substitute for the widely used CdTe and CuInGaSe_2_ materials.^2^ The components of CZTS are cost-effective due to the substitution of indium (In) with low-cost zinc (Zn) and tin (Sn), and they are also environmentally friendly and composed of abundant, earth-derived materials.^3^ CZTS demonstrates a high absorption coefficient (*α* > 10^4^ cm^−1^), a direct band gap between 1.4 eV and 1.6 eV, and exhibits p-type semiconductor characteristics.^4,5^ The significant drawbacks of CZTS chalcogenide-based solar cells include challenges in obtaining single-phase films, controlling the structure of elements, and avoiding the potential existence of ternary and binary secondary phases such as SnS, Cu_2−*x*_S, Cu_2_SnS_3_, and Cu_3_SnS_4_. To resolve these issues, chalcogenide-based solar cell thin films have become a key focus of research because of their favorable physical and chemical properties. The growth of single-phase CZTS is challenging owing to its complex formation and phase diagram, which has a narrow window for structure of single-phase. Additionally, detecting and suppressing secondary phases, such as SnS, Cu_2_S, Cu_2_SnS_3_, and Cu_3_SnS_7_, that form in CZTS thin films under certain processing conditions remains a significant challenge. Multiple approaches involving post-deposition chemical etching have been explored to remove undesired phases from the films.^6^ Single-phase CZTS formation was not attained since chemical etching solely targets surface secondary phases, while the internal phases within the material remain unchanged. Furthermore, because Zn has a higher vapor pressure than Cu, Sn, and S, it is challenging to manage the Zn composition in the manufacturing of CZTS. Zn-induced defects, mainly high-level ‘killer defects’ are significant factors leading to the weak performance observed in CZTS-based solar cells.^7,8^ These phases can significantly affect the performance of photovoltaics. Suppose these secondary phases comprise wide-ranging energy band materials, including ZnS. In that case, they lessen the absorber layer's adequate volume, which possesses the optimal energy gap for single heterojunction photovoltaics.^9^ This decrease subsequently results in a reduction in the short-circuit current. Furthermore, ZnS can form anywhere in the CZTS, enhancing recombination and impeding carrier transit.^10^ To solve these difficulties, obtaining ternary Cu–Sn–S (CTS) samples is more straightforward. Controlling the structure and defects in the ternary Cu–Sn–S system is more concise than quaternary CZTS.

Recently, Cu_2_ZnSnS_4_ (CZTS) and Cu_2_SnS_3_ have attracted considerable attention as photocatalytic materials due to their earth-abundant composition and relatively narrow band gaps. CTS is generally easier to synthesize among these semiconductors than CZTS because it contains fewer elements. For instance, Keerthana *et al.* reported the synthesis of CZTS *via* a simple hydrothermal method, obtaining nanosheet-like morphologies, and indicated that the obtained 0.4 M hexamethylenetetramine-assisted CZTS sample eliminated the RhB dye under visible light with a higher efficiency of 84%.^11^ Similarly, Zhong *et al.* synthesized 3D hierarchical Cu_2_FeSnS_4_ microstructures, achieving 73% degradation of Rhodamine B under visible-light irradiation.^12^ Umbrajkar *et al.* demonstrated that CZTS nanoparticles could efficiently degrade linezolid, reaching 86.97% removal at pH 7, 45 °C, and 120 rpm, with hydroxyl and proton radicals playing a key role; the catalyst maintained high reusability over three cycles, improving degradation efficiency from 49.09% to 80.08%. These findings have inspired us to prepare non-toxic CTS thin films as visible-light-driven photocatalysts. CTS films can exist in multiple crystalline forms, including cubic, tetragonal, triclinic, and monoclinic. Recently, Cu_2_SnS_3_ has drawn much interest in photocatalysts and photodetector applications since CTS, composed of eco-friendly elements and possessing a higher *α* absorption value with a direct energy band gap of 0.95 eV for its cubic form and 1.35 eV for its tetragonal structure. However, there is inadequate literature on adjusting the energy gap value and improving the structural properties of CTS. Rapid industrial development in pharmaceuticals, textiles, and agriculture has brought about environmental problems such as organic pollution, which threatens the environment and human health. Some dyes are used in industries like plastics, food, pharmaceuticals, paper, paints, leather, and textiles.^13^ Methylene Blue (MB), Rhodamine B (RhB), and Methyl Orange (MO) dyes mixed with water are very harmful to human and animal health.^14,15^ MB, a cationic organic dye, limits light diffusion in water and prevents natural purification and photocatalysis. Therefore, MB dye adversely affects aquatic life, especially the plant environment. Radical oxidation processes have attracted much attention from researchers to treat MB dye mixed wastewater containing the MB dye. Photocatalyst, one of the radical oxidation processes that oxidizes all dyes, is a non-toxic, ecological, green, and simple technology.^16^ Photocatalytic degradation offers an efficient and cost-effective method for pollutant removal without causing secondary pollution. TiO_2_ and ZnO semiconductors, widely used in photocatalytic degradation, have the disadvantages of narrow absorption bands, wide band gaps, and low photon utilization. In addition, these oxide semiconductors play an active role in the UV region, constituting 5% of the solar spectrum, which limits the photocatalyst efficiency.^17^ Therefore, semiconductors must respond to light in the visible region, which forms about 45% of the solar spectrum.

The lack of affordable and clean water has been the world's biggest problem due to population explosion and industrialization. To solve this problem, scientists have introduced different photocatalysts using nanotechnology. The most effective photocatalysts are TiO_2_ and ZnO, but these materials are UV-active, making them unsuitable for commercial applications.^18,19^ It has been explored that those semiconductors with narrow energy band gaps can be used as alternatives to photocatalysts like TiO_2_ or ZnO. Copper-based chalcogenides are among the most promising visible light active semiconductors.^20,21^ Cu–Sn–S structure is among the copper-based chalcogenides of photocatalysts. Lately, Umehara *et al.* fabricated Cu_2_Sn_0.83_Ge_0.17_S_3_ and subjected it to a sulfurization procedure. Recently, Umehara and colleagues developed Cu_2_Sn_0_._83_Ge_0_._17_S_3_ thin films and enhanced them through a sulfurization process.^22^ By partially substituting tin (Sn) with germanium (Ge), they could fine-tune the band gap within the range of 0.93 to 1.02 eV and promote grain growth during thermal treatment. Furthermore, they fabricated solar cell devices based on these Ge-doped films, achieving a power conversion efficiency (PCE) of 6.0%. This study demonstrated the effectiveness of targeted cation substitution in optimizing both the electronic and structural properties of Cu_2_SnS_3_ absorbers for improved photovoltaic performance.

The radical scavengers are used to examine the photocatalytic mechanism. The scavengers determine radicals or the main active species performing in a photocatalytic process. Specific scavengers such as ethylenediaminetetraacetic acid disodium salt (EDTA-2Na), propanol (IPA), and *p*-benzoquinone (BQ) added to the system reduce photocatalytic activation, which demonstrates that captured radicals are the primary active species of the hole (h^+^), ˙OH, and ˙O_2_^−^ in the photocatalytic mechanism.^23,24^

The pH value of the solution is a very effective factor affecting the photocatalyst process. pH influences the electrical charge properties on the surface of the catalyst and determines the ionization of the catalyst surface, which involves the degradation of dye compounds. A change in photocatalytic activation can occur as the catalyst and the pollutant will behave differently at different pH levels. The pH value affects the oxidation potential of the catalyst's valence band, the charge dispersion on the catalyst surface, and the decomposition capacity of compounds.^25,26^

The Gd^3+^ ion possesses a comparatively larger ionic radius than Cu^2+^ and Sn^4+^ ions. As a result, the incorporation of Gd into the CTS lattice is anticipated to promote the formation of a solid solution that accommodates Gd^3+^, potentially enabling the synthesis of a Cu–Sn–S–Gd alloy or the development of Cu_2_SnGdS_3_ thin films. The cubic phase of undoped CTS exhibits an optical band gap of approximately 0.95 eV. However, previous studies have demonstrated that doping with Cd increases the optical band gap to around 1.37 eV, while Ge doping results in a band gap of approximately 1.23 eV.^27,28^ These findings suggest that Gd doping will also likely modify the electronic band structure, leading to a distinct shift in the optical band gap relative to the undoped material. In this study, we state the fabrication of Cu_2_Sn_1−*x*_Gd_*x*_S_3_ thin films using a cost-effective and straightforward spin coating method. Research on Cu_2_Sn_1−*x*_Gd_*x*_S_3_ for photocatalytic applications is still early, and many aspects remain largely unexplored. Among the various factors influencing the fabrication of high-quality thin films, the Gd doping ratio is of primary importance and should be systematically examined first. In particular, the Gd doping has a pronounced impact on thin films' microstructural, compositional, and morphological characteristics, photodetector, and photocatalytic properties. The key novelty of this work lies in the outstanding photocatalytic and photodetector performance demonstrated by the fabricated thin films. Unlike most Cu_2_SnS_3_ thin films reported in literature, which are typically prepared *via* vacuum-based techniques and undoped CTS, our approach offers a significant advantage in enhancing photocatalytic efficiency with Gd doping in Cu_2_SnS_3_. Previous studies using spray-coated CTS thin films have shown approximately 90% degradation of MB dye after 3 hours of light exposure, which is relatively slow for practical photocatalytic applications. In contrast, our CTS thin films achieved nearly 90% degradation of MB dye within 90 minutes under similar conditions, highlighting their superior activity. Monoethanolamine (MEA) was used as a capping agent to control particle growth and improve the long-term stability of the CTS sol–gel, thereby extending its shelf life and maintaining its reactivity.

## 2. Experimental

In this study, Cu_2_(Gd:Sn)S thin films were prepared on soda lime glass (SLG) substrates using a precursor solution composed of 0.719 g copper(ii) acetate monohydrate (≥98% purity), 0.542 g tin(ii) chloride dihydrate (≥98% purity), 0.450 g gadolinium(iii) chloride hexahydrate (≥99% purity), and 0.228 g thiourea (CH_4_N_2_S). Each component was separately dissolved in a solvent system of absolute ethanol and glacial acetic acid. The solutions were stirred magnetically at room temperature for 6 hours to ensure homogeneity before film deposition under optimized conditions. Initially, the solution of thiourea as a sulfur source was gradually introduced into the copper(ii) acetate solution. Then, tin(ii) chloride dihydrate was added to the previously mixed thiourea–copper(ii) acetate solution. The resultant combined solutions continued to be stirred at room temperature for one hour to ensure the development of a homogenous white solution. A few drops of triethanolamine were incorporated to fine-tune the pH levels of the final solution. The molar ratios of copper, tin, and sulfur in the resultant solutions were maintained at 2 : 1 : 3. It is important to note that thiourea exhibits volatile characteristics at high annealing temperatures; hence, it was added in two distinct phases to mitigate any potential loss of sulfur.^14^ Varying volumes of gadolinium(iii) chloride solution were added to the thiourea–copper–tin precursor mixture to synthesize Cu_2_SnS_3_, Cu_2_Sn_0.75_Gd_0.25_S_3_, Cu_2_Sn_0.50_Gd_0.50_S_3_, and Cu_2_Sn_0.25_Gd_0.75_S_3_ thin films. Before the deposition process, the soda lime glass (SLG) substrates underwent a two-stage cleaning protocol. Initially, the substrates were boiled at 105 °C in distilled water, ammonia (NH_3_), and hydrogen peroxide (H_2_O_2_). This was followed by a second cleaning step in a mixture of distilled water, H_2_O_2_, and hydrochloric acid (HCl), also maintained at 105 °C under continuous magnetic stirring, to ensure complete removal of surface contaminants. Subsequently, the synthesized SLGs were subjected to a cleaning process using deionized water for three minutes each. After this cleaning step, the samples were dried thoroughly with nitrogen gas to remove residual moisture. Following the preparation of final solutions and the thorough cleaning of SLG substrates, solutions were applied onto SLGs by spin coating at 1500 rpm for 70 seconds under ambient conditions. After cleaning SLGs, final solutions were applied in successive layers, each thermally treated at 220 °C for nearly 10 minutes. After this procedure, samples processed by spin coating were annealed under 50 mg sulfur and an argon atmosphere to avoid the possible oxide phases at 500 °C temperatures for 60 min. The obtained sample names are CTS, CTGS-1, CTGS-2, and CTGS-3 for Cu_2_Sn_1−*x*_Gd_*x*_S_3_ (where *x* = 0), Cu_2_Sn_1−*x*_Gd_*x*_S_3_ (where *x* = 0.25), Cu_2_Sn_1−*x*_Gd_*x*_S_3_ (where *x* = 0.50), and Cu_2_Sn_1−*x*_Gd_*x*_S_3_ (where *x* = 0.75), respectively.

**Fig. 1:**
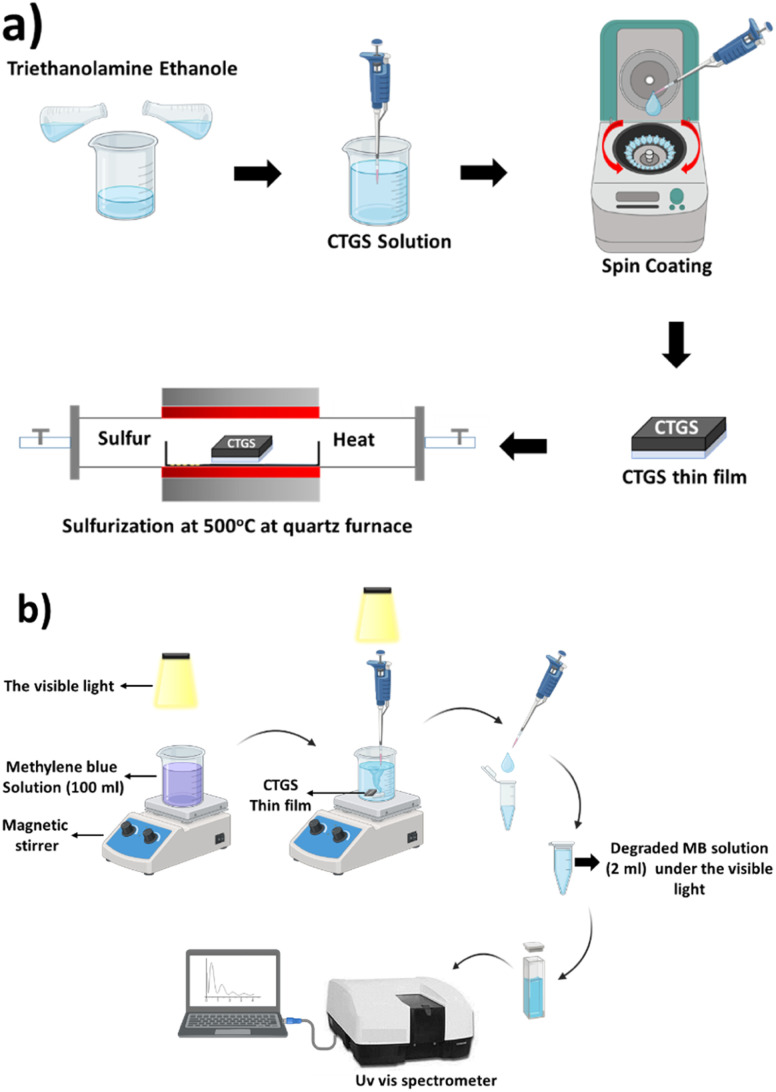
(a) Systematic diagram of spin coating and (b) photocatalyst application process.

This study systematically investigated the effects of Gd doping on the structural, morphological, optical, and photocatalytic properties of the synthesized thin films. Crystallographic analysis was performed using an X-ray diffractometer (XRD, Panalytical/Ampyrean) operated at 40 kV and 40 mA. Measurements were carried out in a *θ*–2*θ* configuration with a step size of 0.02°, enabling detailed evaluation of phase composition and crystallinity. Raman spectroscopy was conducted using a confocal Raman microscope to complement the structural analysis. Surface morphology and elemental distribution were examined *via* field emission scanning electron microscopy (FE-SEM, ZEISS Gemini SEM 500) equipped with an energy-dispersive X-ray spectroscopy (EDX) system. Optical properties, including transmittance and energy band gap, were analyzed using a Shimadzu UV-3600 spectrophotometer (Shimadzu, Tokyo, Japan) over a 300–1100 nm wavelength range. The photodegradation activity of Gd-doped CTS (catalyst) in organic waste solution containing MB dye was studied at room temperature. The first 100 ml of MB dye solution was placed in the container and put on a magnetic stirrer, where the experiment was carried out, as seen in Fig. 1. The absorption peak of the only MB dye solution was determined to be 664 nm wavelength *via* a UV-Vis absorption spectrometer. CTS, CTGS-1, CTGS-2, and CTGS-3 thin films were placed separately in MB dye, each in the dark for 20 minutes. The dye was then taken from the container with a syringe. Then, it was taken from the MB dye wastewater containing these thin films every 20 minutes under visible light using a halogen lamp (250 W metal halide lamp (GE ARC250), 173.8 W m^−2^ light intensity) until the end of 220 minutes.

## 3. Results and discussion

### 3.1. XRD analysis

**Fig. 2:**
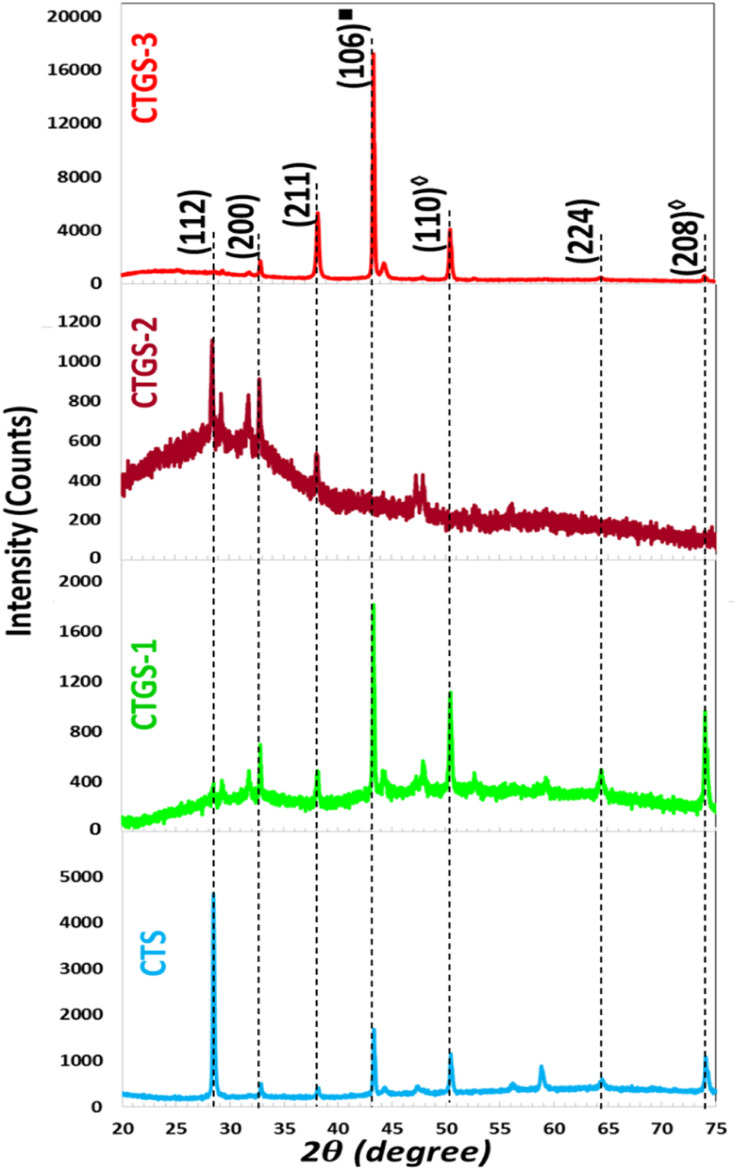
XRD spectra of CTS samples with different Gd doping.

Fig. 2 shows XRD patterns of Cu_2_Sn_1−*x*_Gd_*x*_S_3_ with different Gd and Sn doping ratios deposited on a glass substrate at 550 °C, which are denoted as CTS, CTGS-1, CTGS-2, and CTGS-3, respectively. The intense peak for Cu_2_Sn_1−*x*_Gd_*x*_S_3_ film (where *x* = 0) emerging at 2*θ* = 28.44° corresponds to the Cu_2_SnS_3_ phase. It is seen that changing the Gd/Sn doping ratio changes the position of peaks. A few peaks related to the secondary phases, including CuS and SnS_2_, are also present in the obtained thin film. Cu_2_SnS_3_ was reportedly crystallized in different crystallite structures such as tetragonal, cubic, or monoclinic.^29,30^ According to the reported structural data of Cu_2_SnS_3_, the variation might be caused by different growth temperatures and annealing conditions used for the fabrication processes.

All synthesized films exhibited diffraction peaks near 2*θ* values of 28.44°, 32.64°, 38.28°, 43.20°, 50.29°, 58.92°, and 74.19°.^31^ The intensity of the (106) peak, associated with the hexagonal CuS covellite phase, was notably influenced by the tin concentration in the precursor solution. In the CTGS-2 film, this (106) reflection was absent, likely due to the low melting point of tin, which can result in its volatilization during annealing. The formation of this CuS phase may be attributed to the diffusion of Gd within the alloy matrix and the preferential formation of Cu–S bonds, accompanied by the displacement or loss of Sn. From X-ray diffraction data, the positions of peaks for the CTS film shifted slightly with increasing Gd/Sn dopant. Peak intensity, broadening, and shift indicate that Gd^3+^ ions are successfully embedded into the CTS lattice. The intensity of the (112) cubic CTS peak decreased due to a decrease in the Gd/Sn ratio. This indicates that the lattice structure suffers dilation. The main intensity peak for CTS thin film displayed a major peak at 2*θ* = 28.44° corresponding to (112) plane with a slight shift to a higher angle with adding Gd doping in solution where Gd prefers to substitute Sn site in CTS lattice because of large effective ionic radius of Gd^3+^ compared to Cu^2+^, Sn^2+^ as small ions were replaced with large ions. Specifically, the effective ionic radii of Gd^3+^, Cu^2+^, and Sn^2+^ are 0.94 Å, 0.74 Å, and 0.69 Å, respectively. The formation of the secondary phases acts as a source of structural defects, disrupting the long-range order of the CTS lattice and resulting in non-uniformity within the thin films. Such structural irregularities cause local distortions and induce compositional fluctuations, adversely affecting the film's peak position and growth. Moreover, these defects lead to variations in the energy band alignment across different material regions, thereby introducing localized states and potential barriers. Magdy *et al.* reported that the intensity of the peak exhibited (112) preferential orientation, with a shift to a higher angle attributed to antimony (Sb) doping, where Sb predominantly substitutes the Sn site in Cu_2_SnS_3_.^32^

The inter-planar spacing values (*d*_*hkl*_) the thicknesses of tetragonal CTS thin films were calculated using XRD measurements based on Bragg's law, given in eqn (1), and presented in Table 1.

1 2*d*_*hkl*_ sin *θ* = *nλ*

**Table 1:** Crystallite parameters of CTS, CTGS-1, CTGS-2, and CTGS-3 thin films

Sample	2*θ*	*β* (radian)	*D*_*hkl*_ (nm)	*d*_*hkl*_ (Å)	*δ*_*hkl*_ (×10^14^ m^−2^)	*N*_*hkl*_ (×10^15^ m^−2^)	*ε*_*hkl*_ (×10^4^)	*hkl*
CTS	28.45	0.0027	55.33	3.315	3.267	7.1	26.63	(112) Cu_2_SnS_3_
	43.30	0.0036	43.28	2.088	5.339	14.8	22.67	(106) CuS
	50.46	0.0036	44.47	1.807	5.058	13.6	19.10	(110) SnS_2_
	58.97	0.0059	28.20	1.565	12.579	53.5	26.09	(228) Cu_2_SnS_3_
	74.12	0.0036	50.41	1.278	3.936	9.4	11.92	(208) CuS
CTGS-1	32.79	0.0028	54.10	2.729	3.416	7.6	23.71	(200) Cu_2_SnS_3_
	38.24	0.0036	42.57	2.352	5.517	15.6	25.96	(211) Cu_2_SnS_3_
	43.30	0.0031	50.26	2.088	3.959	9.5	19.52	(106) CuS
	50.39	0.0041	39.03	1.809	6.564	20.2	21.79	(110) SnS_2_
	74.07	0.0031	58.52	1.279	2.920	6.0	10.57	(208) CuS
CTGS-2	28.44	0.0029	51.51	3.136	3.769	8.8	28.61	(112) Cu_2_SnS_3_
	32.78	0.0028	54.10	2.730	3.417	7.6	23.71	(200) Cu_2_SnS_3_
	38.16	0.0038	40.32	2.356	6.151	18.3	27.47	(211) Cu_2_SnS_3_
CTGS-3	32.92	0.00314	48.09	2.718	4.324	10.8	26.56	(200) Cu_2_SnS_3_
	38.20	0.0052	29.47	2.357	11.517	46.9	37.58	(211) Cu_2_SnS_3_
	43.37	0.0032	45.83	2.085	4.760	12.5	21.38	(106) CuS
	50.49	0.0043	37.23	1.806	7.214	23.3	22.80	(110) SnS_2_

The crystallite size (*D*_*hkl*_) is computed by Scherrer's formula based on the relation given in eqn (2).^33^2
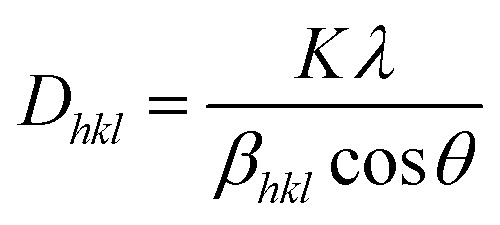
*λ* is the wavelength of the XRD measurement system, *θ* is Bragg's diffraction angle, and *β*_*hkl*_ is the value of full width at half maxima (FWHM). The *D*_*hkl*_ (112) peak varies from 28 to 58 nm as Gd doping decreases in the solution. This change is because of the partial dissociation of Cu_2_SnS_3_ related to the change in Gd doping. A similar pattern has previously been documented in related thin film studies.^34,35^ The variation in crystallite size may be attributed to an increase in crystallographic defects and lattice mismatch between the host matrix and dopant ions. This trend is further corroborated by particle size estimations derived from FE-SEM images, which align with the observed changes in crystallite dimensions. The narrowing of diffraction peaks is indicative of nanocrystal formation. In contrast, the prominent central peak in the spectra confirms the crystalline nature of the synthesized CTS films, characterized by diverse crystal orientations.

The dislocation density (*δ*_*hkl*_) quantifies the number of dislocations in the crystal lattice, while the micro-strain (*ε*_*hkl*_) indicates the local strain introduced by defects. The number of crystallites (*N*_*hkl*_) corresponds to the number of individual crystalline regions. *t* is the film thickness. The values of the *δ*_*hkl*_, *ε*_*hkl*_, and *N*_*hkl*_ of the obtained films are calculated using the formulas given in eqn (3) through eqn (5).^36^3
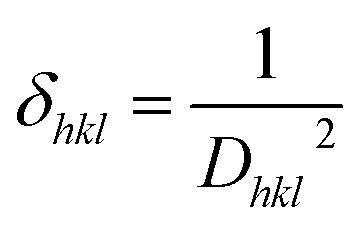
4
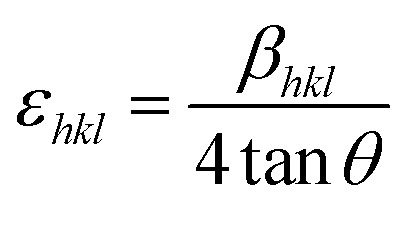
5
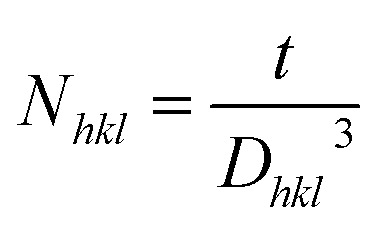


The values of *δ*_*hkl*_, *ε*_*hkl*_, and *N*_*hkl*_ the obtained films grown on SLGs at an annealing temperature of 500 °C for 60 min under a sulfur atmosphere are also displayed in Table 1. The *D*_*hkl*_ for (112) peak decreases, while the *δ*_*hkl*_, *ε*_*hkl*_, and *N*_*hkl*_ increase in CTS compared to the other thin films, indicating an increase in lattice imperfections. This suggests that Gd dopant ions have effectively substituted CTS, resulting in crystallographic defects and changed structural properties. In crystal structures, micro-strain presence typically leads to physical defects and dislocations. Therefore, the extent of such defects is closely linked to the internal strain within the lattice.

### 3.2. Raman

**Fig. 3:**
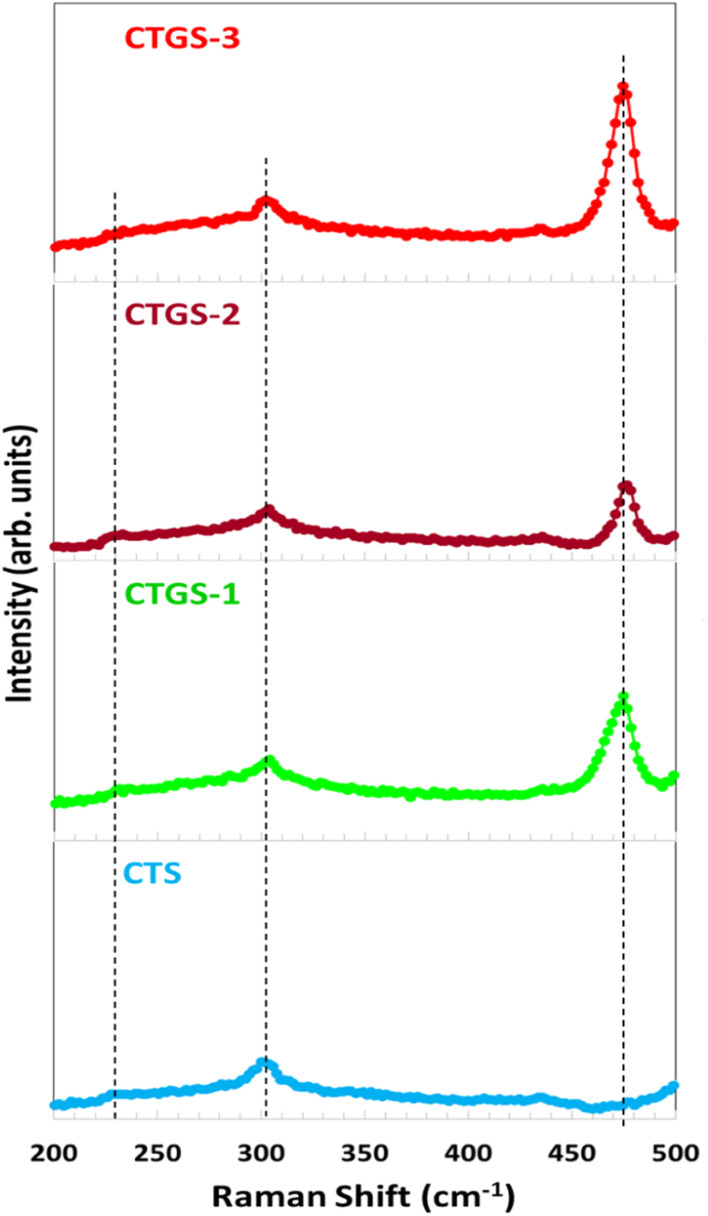
Raman spectra of Cu_2_Sn_1−*x*_Gd_*x*_S_3_ thin films with varying Gd doping concentrations.

Previous studies on Cu_2_SnS_3_ thin films have reported the existence of secondary phases such as binary Cu–S and Sn–S compounds, as well as ternary phases like Cu_4_SnS_4_ and Cu_2_Sn_3_S_7_.^37–39^ Identifying a pure CTS phase is particularly challenging using XRD alone due to the structural similarities among various Cu–Sn–S phases. Therefore, this work employed Raman spectroscopy to perform a more detailed structural analysis of CTS films, enabling better differentiation between the coexisting phases. Fig. 3 demonstrates Raman spectra of samples with different Gd doping in solution. Some minor variations in frequency could be attributed to differences in composition, growth parameters, and the microstructural properties of CTS thin films. We have observed a low-intensity Raman peak at 229 cm^−1^, which may be attributed to amorphous SnS_2_.^40,41^ This film shows a peak in the wavenumbers of 302 cm^−1^, which also denotes Cu_2_SnS_3_ in the cubic phase.^42^ These vibrational characteristics are in good agreement with the data presented by Fernandes *et al.*,^43^ who reported that Raman analysis was obtained and linked to XRD data, permitting the assignment of the peak at 303 cm^−1^ to a cubic phase of Cu_2_SnS_3_.

All spectra show a peak around 476 cm^−1^, representing CuS covellite Raman mode.^44^ Increasing the Gd doping in solution resulted in slight shifts in Raman peaks, likely due to stoichiometric variations caused by the volatilization of tin (Sn) and sulfur (S) at higher thermal conditions. Elemental Sn has a melting point of 232 °C, and sulfur has a melting point of 110–120 °C.^45,46^ During the heating stage of the sulfurization process, the precursors are expected to transition into a Cu partially-Sn metallic liquid phase, which subsequently reacts with sulfur to form SnS, CuS, and Cu_2_SnS_3_ phases. Precursors with a higher Sn content tend to favor the formation of SnS to a greater extent. However, because of the high volatility of the SnS phase at higher temperatures, the loss of elemental Sn is likely to occur through the decomposition and evaporation of SnS from the precursor films.^47^

### 3.3. Morphological properties

**Fig. 4:**
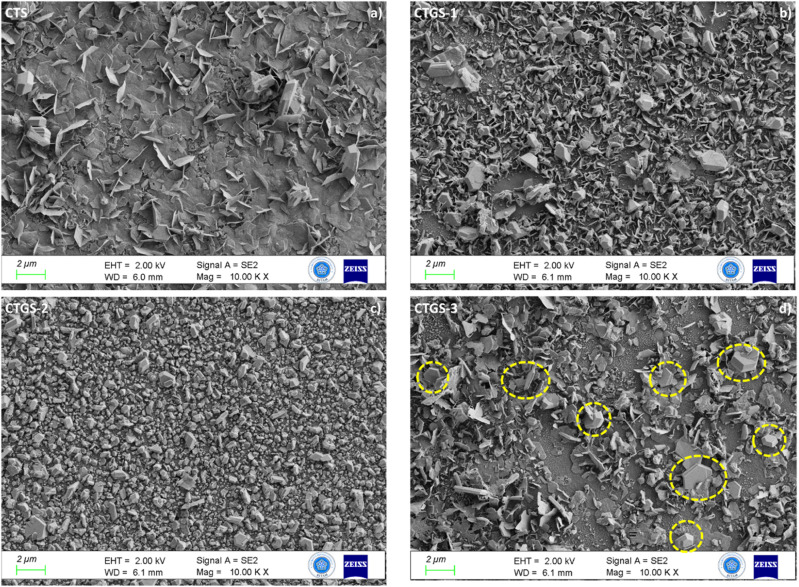
FE-SEM images of (a) CTS, (b) CTGS-1, (c) CTGS-2, and (d) CTGS-3 thin films.

FE-SEM analysis provides critical insight into the morphological characteristics of the fabricated thin films. Variations in the Gd/Sn ratio lead to notable alterations in surface morphology, as illustrated in Fig. 4. The figure shows films deposited on SLGs using the spin coating technique, followed by sulfurization at 500 °C in a sulfur and argon environment in a quartz furnace. In the case of Cu_2_Sn_1−*x*_Gd_*x*_S_3_, lower Gd concentrations during film preparation have been correlated with the formation of smaller grains. An increase in the Gd/Sn doping ratio leads to some development of larger grain structures, indicating enhanced grain growth due to higher dopant concentration. Still, it does not entirely cover the surface of the thin film. The resulting microstructures predominantly exhibit spherical grains and polygonal formations indicated in a circle. FESEM analysis reveals the formation of well-ordered sheet-like structures that are oriented nearly perpendicular to the SLG substrate. This two-dimensional, sheet-like morphology is widely recognized as an intrinsic structural characteristic of SnS_2_.

In contrast, Cu_2_SnS_3_ and CuS thin films generally exhibit a different growth behavior, where the grains aggregate into large clusters of varying sizes. Such morphological changes ultimately affect their optical and electronic properties. However, a significant morphological transformation is observed with increasing Gd concentration and higher annealing temperatures in a sulfur-rich atmosphere. The emergence of larger clustered structures and surface feature transitions marks this transformation. The films progressively develop a densely packed grain configuration with nanosheet- or flake-like textures, indicating that thermal treatment and dopant concentration influence nucleation dynamics and surface uniformity.^31^

Sayed *et al.* indicated that Ge-doped Cu_2_SnS_3_ thin film showed a grain structure larger than the undoped Cu_2_SnS_3_ thin film due to Ge incorporation into the Cu_2_SnS_3_ layer.^48^ Chalapathi *et al.*^49^ reported that as the atomic percentage of Sb increased, the diffusion of Sb atoms toward the film's surface intensified, promoting larger, compact, and well-defined round-shaped grains.^49^ Notably, both intact and fragmented nanosheet structures are discernible in the obtained thin films sulfurized at 500 °C, as seen in Fig. 4. The FE-SEM micrographs reveal a comprehensive overview of the surface, characterized by tightly packed polygonal grains and compact nanosheet assemblies. The aggregation of Cu_2_Sn_1−*x*_Gd_*x*_S_3_ nanoparticles promotes the growth of homogenous grains. At high Gd concentrations, enhanced coalescence of crystalline grains is evident, resulting in larger particle sizes and improved structural uniformity. Consequently, Gd doping levels are associated with reduced charge carrier concentrations compared to Cu_2_SnS_3_ thin film and narrowed energy band gaps, contributing to enhanced optoelectronic device performance.

**Fig. 5:**
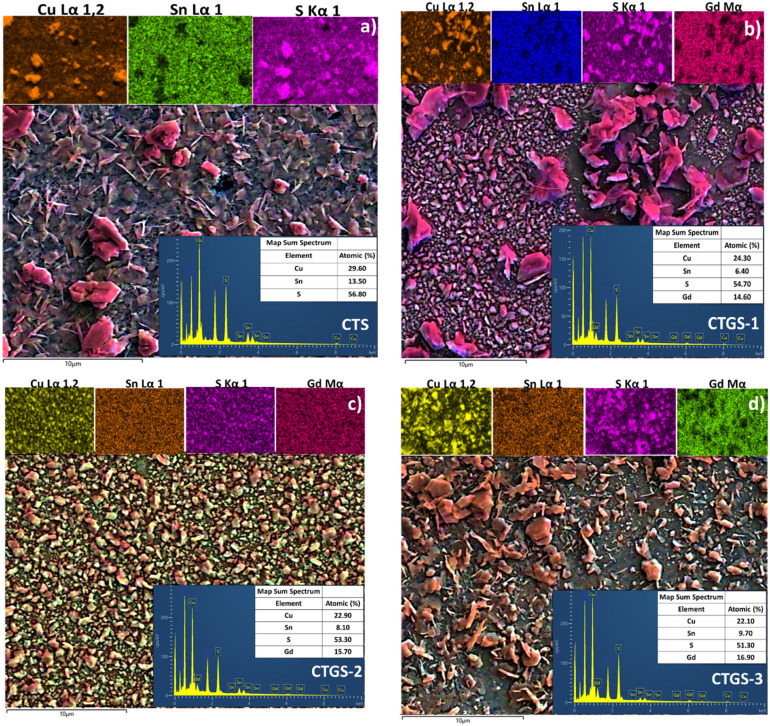
EDX mapping images of (a) CTS, (b) CTGS-1, (c) CTGS-2, and (d) CTGS-3 thin films.

Fig. 5a–d displays the atomic composition data for CTS, CTGS-1, CTGS-2, and CTGS-3 thin films. As illustrated, all samples exhibit a tin-deficient profile, which aligns with the targeted stoichiometry. Cu/Sn atomic ratios for CTS, CTGS-1, and CTGS-2 vary between 2.19 and 3.80, suggesting that an increased Gd/Sn doping ratio may contribute to Sn depletion during synthesis. The calculated S/(Cu + Sn + Gd) atomic ratios are 1.32 for CTS, 1.21 for CTGS-1, 1.41 for CTGS-2, and 1.05 for CTGS-3. These results indicate a surplus of sulfur relative to the ideal stoichiometric composition of CTS. This sulfur excess is likely due to the slow cooling process inside the sealed quartz furnace, which allows continued sulfur incorporation after the high-temperature reaction phase.

### 3.4. Optical properties

**Fig. 6:**
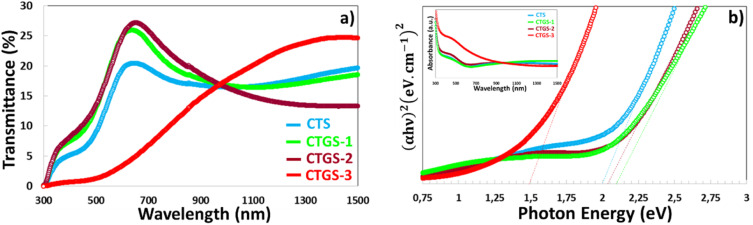
(a) Transmittance, (b) energy gap of CTS, CTGS-1, CTGS-2, and CTGS-3 thin films.

Transmittance (*T*%) graphs are presented in Fig. 6a. The CTS sample shows an increase in transmission compared to CTGS-1 and CTGS-2 thin films in the visible and near-infrared (IR) regions, and transmittance reached 27% after adding Gd doping. CTS, CTGS-1, and CTGS-2 thin films display a transmittance value of around 20–27% in UV-Vis ranges of spectra that reduces in the region close to 664 nm; also, CTGS-3 thin film exhibits low transmittance (∼1%) in the lower wavelength range, which gradually increases to approximately 22% in the IR region. This behavior may be attributed to the degeneration of semiconductor characteristics in the film and the existence of secondary phases, including CuS and SnS_2_ phases.^50^ The observed trend seems to be due to changes in the crystalline quality of the Cu_2_SnS_3_ phase. The films contain absorption centers in the energy band, contributing significantly to photon absorption. These effects are caused by defects like vacancies and interstitial substitutions.^51^ The variation in transmittance of CTS thin films can be significantly influenced by the formation of secondary phases, which often arise from non-stoichiometric compositions or incomplete reactions during fabrication. Phases such as CuS or SnS_2_ can act as scattering centers or additional absorption sites, thereby modifying the overall optical transmittance. Furthermore, doping may induce defects or additional secondary phases, which further enhance light absorption and scattering, emphasizing the importance of controlling phase purity and defect density to optimize the optical performance of CTS films.

The optical energy band gap (*E*_g_) is a key parameter for photovoltaic (PV) absorber materials. In this study, *E*_g_ with the addition of Gd doping, the value of the CTS thin film was determined using UV-Vis spectrophotometry based on transmittance measurements. As depicted in Fig. 6b, by plotting (*αhν*)^2^ against *hν* and extrapolating the linear region, the direct *E*_g_ was estimated to be approximately 2.0, 2.05, 2.10 eV, and 1.50 eV for CTS, CTGS-1, CTGS-2, and CTGS-3 thin films, respectively. The differences observed in the energy band gap values can be primarily attributed to stoichiometry variations and secondary phases^31^ seen in the Raman and EDX. In particular, CTS thin films annealed at higher temperatures in a quartz furnace tend to suffer from Sn evaporation due to the low melting point of Sn. This Sn deficiency condition favors the formation of secondary CTS-related phases.^31^ Compared to the well-accepted band gap value of ∼1.4 eV for stoichiometric CTS films,^52^ the higher values obtained in this study and the lower value of 1.50 eV can be explained by the coexistence of SnS_2_ and CuS secondary phases, as confirmed by the XRD analysis. In addition, we observed that the XRD pattern of the CTGS-3 film presented maximum intensity secondary CuS phase, which acts as defects and introduces energy levels within the bandgap, reducing the bandgap energy. Secondary phases in CTS (copper tin sulfide) thin films are generally regarded as defect states, which can significantly influence the material's optical and electronic properties, including its band gap. The influence of such secondary phases on the optical characteristics of CTS films has also been previously documented in literature.^52,53^ Patel *et al.*^54^ stated that the energy gap of Cu_2_SnS_3_ thin films differs between 1.29 eV and 1.73 eV with the absorption coefficient (*α*) of >10^4^ cm^−1^, depending on the copper concentration, based on samples synthesized *via* the spray pyrolysis technique. Similarly, Chaudhari *et al.*^55^ studied the effect of the sulfurization process. They observed a band gap range of 1.01 to 1.45 eV for as-grown and sulfurized Cu_2_SnS_3_ thin films *via* the spin-coated method. Shelke *et al.*^56^ calculated an energy band of 1.31 eV for as-deposited CTS films, which increased to 1.35 eV following post-deposition annealing, using the chemical bath deposition (CBD) method. These findings collectively indicate that the optical band gap of CTS thin films is highly sensitive to the synthesis method and processing conditions. However, the relatively narrow *E*_g_ value of Gd:CTS, compared to the optimal energy band gap 1.7 eV for Vis light photocatalysis applications,^57^ indicates that further tuning, such as through gadolinium (Gd) doping, may be required to enhance the efficiency of Gd:CTS-based photodetector and photocatalysis applications.

### 3.5. Photocatalyst mechanism

In this study, we used CTS, CTGS-1, CTGS-2, and CTGS-3 thin films as catalysts to degrade MB dye, known as organic waste. It is seen that MB dye is almost not degraded under visible light without using any catalyst in the graph of Fig. 1. The following equation calculates the degradation efficiency of MB eqn (6):^58,59^6
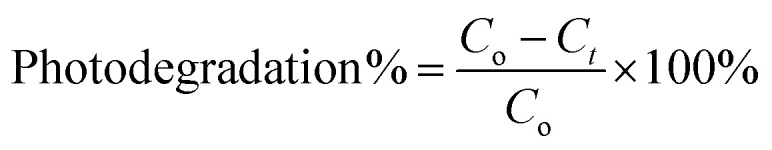
Here, *C*_o_ defines the concentration of MB dye at a time (*t*) = 0 before the catalytic process. *C*_*t*_ represents the value of the MB concentration at the end of *t* time duration. Fig. 1 shows the effect of the Gd ratio in CTS thin film on the photodegradation of MB dye. During the photocatalyst process until the end of 220 minutes, the dye solution was taken from the container every 20 minutes. The photodegradation efficiency of CTS, CTGS-1, CTGS-2, and CTGS-3 thin film catalysts in removing MB dye at the end of 220 minutes (for pH = 10), which was calculated to be 86.16%, 84.37%, 80.76% and 90.77%, respectively. 3% Gd^3+^ doping rate in CTS material, which caused the highest photo-degradation efficiency (90.77%), enabled the high-performance photocatalyst process with an effective electron–hole (e^−^–h^+^) pair separation in CTS material. The particle size increased with some increase in Gd^3+^ ratio; thus, the particles forming the thin film were in greater contact with the MB dye solution with larger surface areas.^24^ Therefore, the reactions of radicals occur at a higher rate with high amounts of charge carriers collected on the material surface. Furthermore, the ionic radius of the Gd^3+^ element (0.94 Å) is larger than the ionic radii of the Cu^2+^ (0.74 Å) and Sn^2+^ (0.69 Å) elements in the CTS, and the Gd atom replaces these atoms, enlarging the crystal size and improving the crystal structure. Since the crystal structure is more developed, the lifetime of charge carriers in CTS is higher, and more photo-excited charges do not recombine inside the traps, which interact with the MB dye solution. In addition, CTGS-3 has a lower band gap compared to others and has high photon absorption in the visible region. More photoexcited charge carriers by the light in the Vis region formed in this thin film act as catalysts.^24,60^ These charge carriers enhance the reaction with radicals. However, CTS, CTGS-1, and CTGS-3 thin films containing CuS and SnS_2_ second phases have lower photon transmission and lower band gaps in the visible region. Thus, the second phase within the thin films can increase photon absorption, forming more photoexcited charge carriers within the semiconductor and improving photocatalyst efficiency. As a result, the CTGS-3 thin film catalyst showed the highest photocatalyst performance by 90.77% in degrading the MB dye solution with all these factors.

CTS, a p-type semiconductor, is formed from a majority of positive charges and is, therefore, suitable for oxidizing organic compounds such as MB dye. The photocatalyst process involves holes (h^+^), hydroxyl (OH), and superoxide ion (O_2_^−^) radicals for the reaction. Vis light is first directed onto a thin CTGS-3 film in MB dye solution to degrade the dye. Light excites electrons in the CTS's valence band (VB) and jumps them to the conduction band (VB), forming e^−^–h^+^ pairs as seen in Fig. 8 (ref. 60) and in eqn (7). These charge carriers move to the surface of Gd-doped CTS catalyst and carry out redox reactions with other species.

The decomposition process of MB dye with CTS semiconductor is expressed by eqn (7)–(12):^59,60^

7 Gd:CTS + *hν* → h^+^ + e^−^

8 h^+^ + H_2_O → OH* + H^+^

9 h^+^ + OH^−^ → OH*

10 e^−^ + O_2_ → O_2_^−^*

11 H_2_O + O_2_^−^* → H_2_O_2_ → 2OH*

12 MB + OH* → degraded dye

h^+^ charge reacts with OH^−^ hydroxyl or H_2_O water molecule to generate free OH* radical (in the eqn (8) and (9)). e^−^ charge reacts with O_2_, which forms O_2_^−^* superoxide in eqn (9). O_2_^−^* reacts with the water molecule, which allows it to form H_2_O_2_ peroxide and then free OH* radical in eqn (10) and (11). The OH* radical produced due to the specific photocatalyst process mentioned above is highly oxidative. It allows the degradation of the dye molecule.^59^ So, the high Gd^3+^ content resulted in enhanced photocatalytic degradation. Thus, when Gd^3+^ ions are doped into CTS, they act as traps for electrons in the conduction band due to their 4f electron configuration, preventing recombination with holes in the valence band. Thus, the content of Gd^3+^ ions increases the amount of hydroxyl radicals by enhancing electron–hole separation and improving photocatalytic activity.^61–63^ All these processes are clearly shown in the band diagram of Gd:CTS in Fig. 8.

**Fig. 7:**
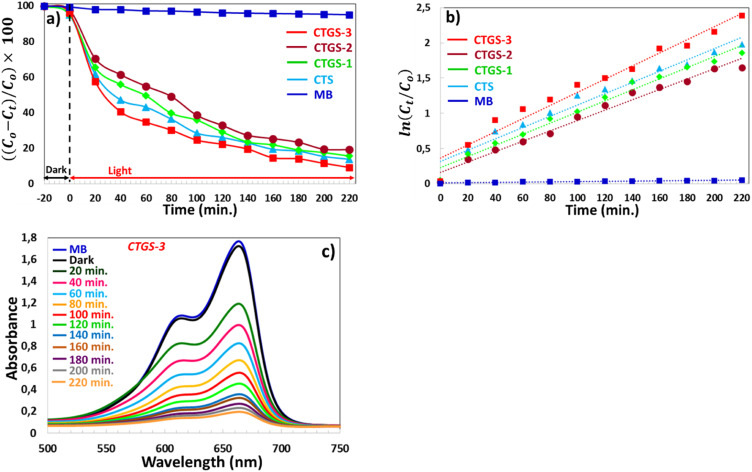
(a) Relative concentration change of MB solution under visible light irradiation using the CTGS-3 photocatalyst at fixed time intervals. (b) Pseudo-first-order kinetic plot for the photocatalytic degradation of MB under visible light using CTGS-3. (c) UV-Vis absorption spectra of MB solution over time under visible light exposure in the presence of the CTGS-3 photocatalyst.

As seen in Fig. 7c and 3, MB dye without a catalyst is very dark blue and shows a high absorption peak around 664 nm wavelength at 0 min. The MB dye containing CTGS-3 catalyst (exhibiting the highest photodegradation efficiency) has undergone photodegradation due to the reaction mentioned above. Thus, the light absorption amount of the dye solution decreased significantly, and the color of the dye became transparent at the end of 220 min.

**Fig. 8:**
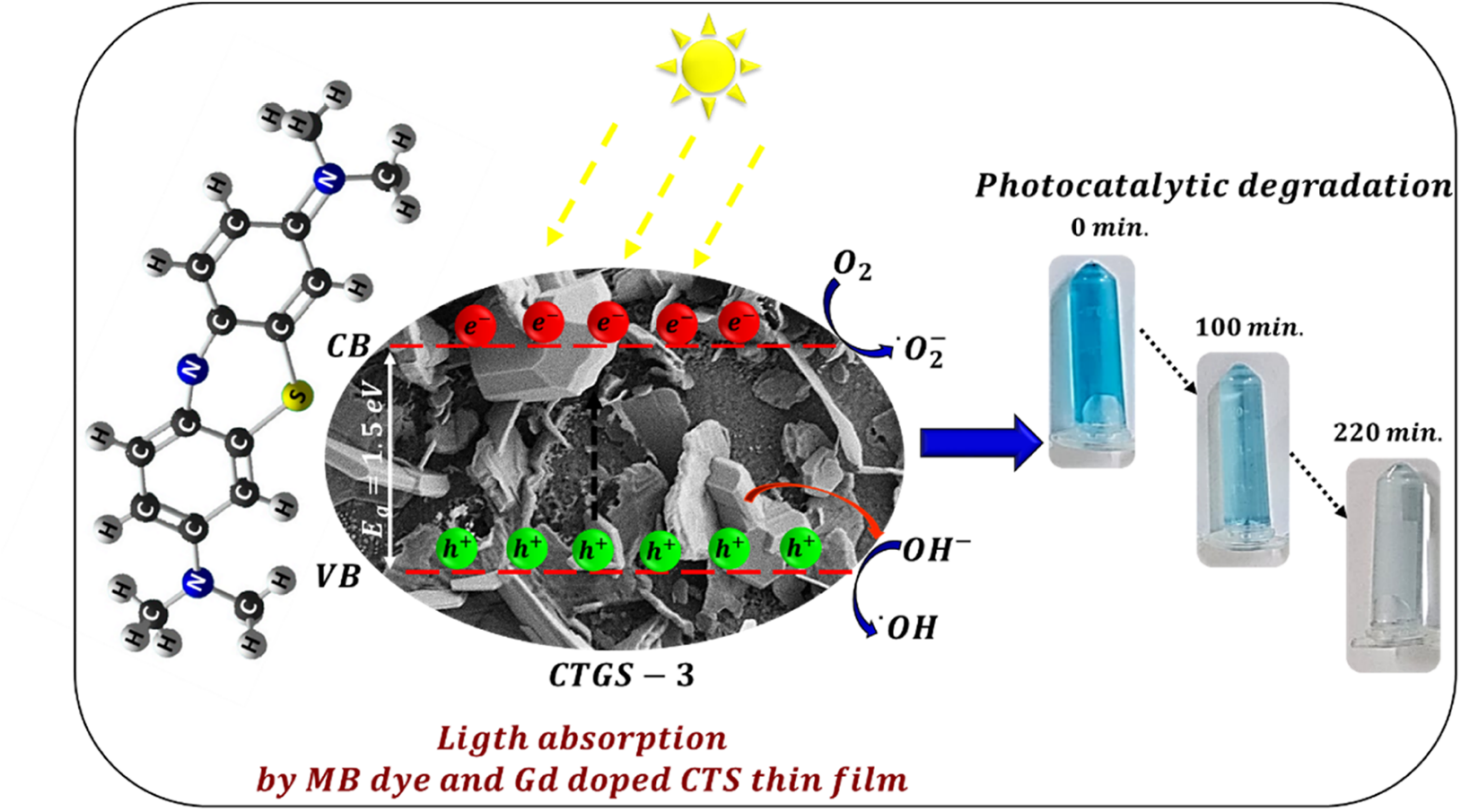
Schematic illustration of the photocatalytic degradation process of MB dye solution using the CTGS-3 thin film under visible light irradiation.

The degradation rate of MB dye is calculated using the model expressed by the Langmuir–Hinshelwood first-order kinetic model in eqn (13):^64^13
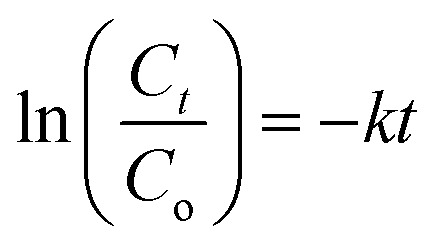
Here, *k* and *t* are the degradation rate constant and time interval, respectively. The first-order degradation reaction of MB dye is shown in Fig. 7b. The calculated degradation rate constant (*k*) of MB blue used in all experiments without any catalyst (for pH = 10) is 0.0002. The linear gradient of Gd-doped CTS thin film catalysts indicates that MB dye degradation occurs following the first-order kinetic model. The rate constant (*k*) of CTS, CTGS-1, CTGS-2, and CTGS-3 thin films catalyst are 0.008 min^−1^, 0.0079 min^−1^, 0.0073 min^−1^ and 0.093 min^−1^, respectively. CTGS-3 catalyst, which exhibits the highest photodegradation, shows the highest degradation rate of 0.093 min^−1^.

**Fig. 9:**
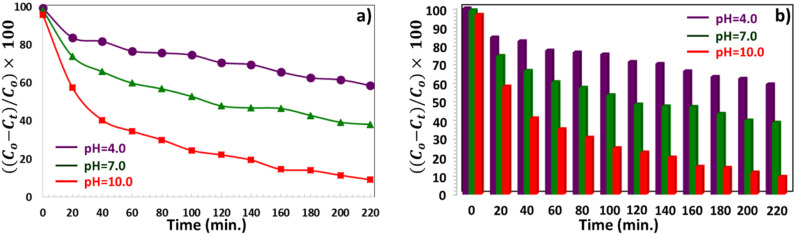
(a and b) Effect of pH on the concentration change of MB solution under visible light irradiation using the CTGS-3 photocatalyst at a fixed time interval.

The pH of the solution considerably affects the photo-degradation process and the kinetic adsorption of dyes using a thin semiconductor film. This study used three different pH values to remove the MB dye. The pH values of the MB solution were adjusted to 4.0 (acidic condition, using 0.1 M HCl), 7.0 (neutral condition), and 10.0 (basic condition, using 0.1 M NaOH). It was investigated that the effect of Gd-doped CTS catalyst on the degradation and adsorption of MB solution at three pH values until 220 min is shown in Fig. 9a and b. According to these figures, for the CTGS-3 catalyst, the photodegradation of MB dye solutions at pH = 4, 7, and 10 was obtained as 41.25%, 61.94%, and 90.77%, respectively. The photocatalyst surface can be negatively or positively charged depending on whether the solution is alkaline or acidic.^25^ The lowest pollutant adsorption takes place as the solution approaches the isoelectric level. The photocatalyst surface becomes positively charged below the isoelectric level and negatively charged above the isoelectric level. More effective electrostatic interaction occurs between MB cations and the negatively charged surface of the photocatalyst for the dye solution at pH = 10. Thus, the degradation of MB dye occurs with faster kinetics.^23,24^ Furthermore, at high pH values, corrosion of photocatalysts is negligible, allowing easier oxidation of the sulfur-containing Gd:CTS component.^25^

**Fig. 10:**
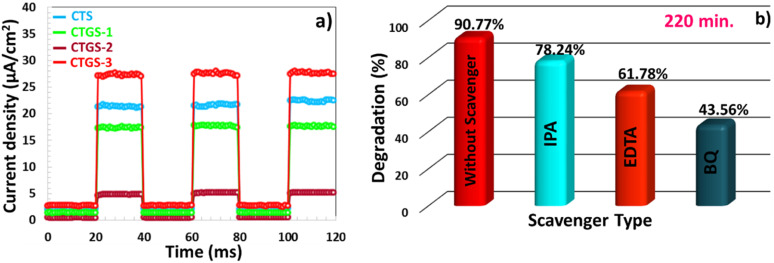
(a) Photocurrent density response of the CTS thin film under visible light irradiation using on/off light cycles. (b) Influence of CTGS-3 photocatalyst on the degradation of methylene blue (MB) solution under visible light in the absence and presence of scavenger agents (IPA, EDTA, and BQ).

Fig. 10 shows the current density graphs depending on time under Vis light on/off exposure cycles for CTS, CTGS-1, CTGS-2, and CTGS-3 photocatalysts.^65^ Photocurrent values predict the amount of separation of e^−^–h^+^ pairs excited in the photocatalyst under Vis light.^23,66^ Among all these catalysts that are responsive to light, CTGS-2 catalyst has the lowest photocurrent (4.9 μA cm^−2^), and the CTGS-3 catalyst has the highest photocurrent (27.55 μA cm^−2^). Moreover, compared with the photocurrent of CTS (21.30 μA cm^−2^), the photocurrent of CTGS-3 is 1.3 times, and the e^−^–h^+^ pair separation is improved in this photocatalyst, contributing more to photodegradation.

Scavengers are radicals that play an essential role in the degradation of waste solutions.^60,65^ In this study, the effect of ethylenediaminetetraacetic acid disodium salt (EDTA-2Na, 5 mmol L^−1^), (IPA, 5 mmol L^−1^), and *p*-benzoquinone (BQ, 1 mmol L^−1^) chemicals that can detect holes (h^+^),˙OH, and ˙O_2_^−^ radical scavengers on the photodegradation (under Vis light) of Gd doped CTS catalyst.^23,24^ The photodegradation efficiency of the MB solution for CTGS-3 is 90.77% without using any scavengers. In the presence of a BQ scavenger, the lowest photodegradation was observed with 43.56% efficiency, indicating that ˙O_2_^−^ radical was very effective in photocatalyst work, as presented in Fig. 5b. The effect of h^+^ radical on photodegradation was slightly lower compared to ˙O_2_^−^, and MB solution exhibited 61.78% photodegradation in the presence of EDTA-Na scavenger. The degradation efficiency of 78.24% obtained in the presence of IPA is close to the efficiency achieved without using any scavenger. As a result, it is seen that ˙OH radical is a scavengers with the least effect on degradation compared to other types of scavengers.

**Fig. 11:**
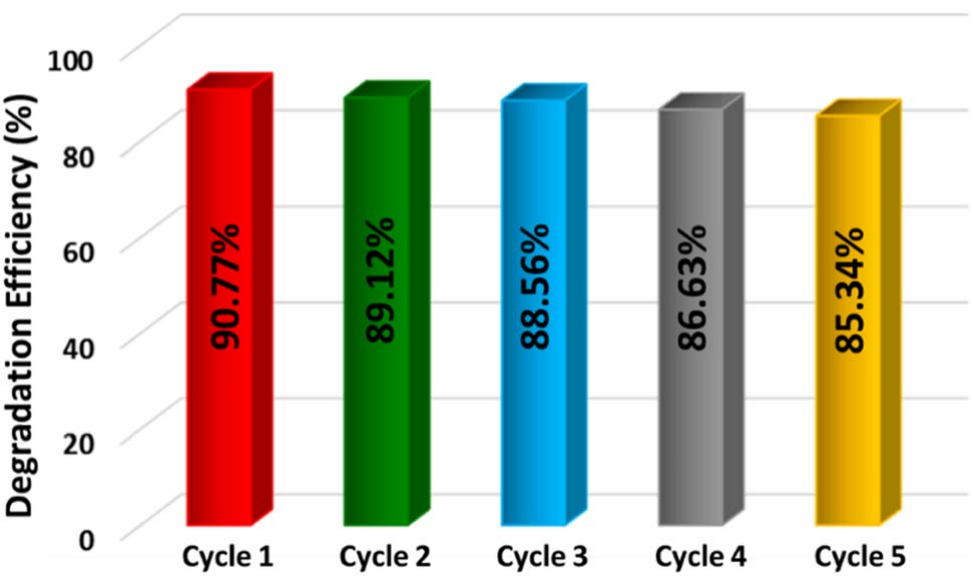
Photocatalytic stability test of CTGS-3 for five recycle.

The stability and reusability of the CTGS-3 photocatalyst are essential for its practical use. Therefore, the photocatalyst was subjected to five consecutive recycle tests as shown in Fig. 11. As a result of five cyclic studies, the photoactive property of the photocatalyst was preserved, but its photodegradation efficiency was slightly reduced with the number of cycles. During the washing process, degradation products can block the active sites on the photocatalyst surface or the photocatalyst can suffer serious permanent loss during the recycling process.^67^

There are some photocatalyst studies based on CTS material in the literature. Comparative values of degradation time, degradation percentage, and degradation rate constant of CTS photocatalysts in these studies and our study are given in Table 2. This research has potential applications in photodegrading harmful pollutants in industrial wastewater and domestic water supplies.

**Table 2:** The result values of the photocatalyst studies based on the CTS material

Sample	Time	Degradation percentage	Degradation rate constant (*k*)	Production technical	References
Cu_2_SnS_3_ nanoparticle	60 min	94.0%	3.5 × 10^−2^ min^−1^	Heat-up method (co-thermolysis)	64
Cu_3_SnS_4_ nanoparticle	60 min	73.0%	1.1 × 10^−2^ min^−1^	Heat-up method (co-thermolysis)	64
Cu_2_SnS_3_ nanoparticle	80 min	80.0%	1.13 × 10^−3^ min^−1^	Green hydrothermal method	68
Cu_2_SnS_3_ bimetallic nanoparticles	60 min	97.57%	65.87 × 10^−3^ min^−1^	Microwave-assisted pathway	69
Cu_2_SnS_3_ thin film	90 min	85%	—	Spin coating sol–gel	57
Cu_2_SnS_3_ thin film	180 min	∼90%	0.01296 min^−1^	Ultrasonic spray pyrolysis	70
Cu_2_SnS_3_ nanostructure	180 min	92%	0.007 min^−1^	Template-free hydrothermal	14
Cu_2_SnS_3_ nanostructure	120 min	95%	—	Hot injection method	71
Flower-like Cu_2_SnS_3_/reduced graphene oxide hybrid material	210 min	87%	—	Hydrothermal method	66
ZnS/Cu_2_SnS_3_ heterojunction	240 min	90.9%	0.6494 h^−1^	Hydrothermal method	72
Flower-like Cu_2_SnS_3_ nanoparticle	90 min	95%	0.03393 min^−1^	Ball milling and the solvothermal method	60
Z-type Cu_2_SnS_3_/g-C_3_N_4_ heterojunction	100 min	99.3%	0.06289 min^−1^	Solvothermal	73
Cu_2_SnS_3_ nanoparticle	150 min	90%	0.0026 min^−1^	Hydrothermal method	13
AgIO_3_/Cu_2_SnS_3_ S-scheme nanoheterostructured	60 min	93.5%	0.15 min^−1^	Hydrothermal method	67
Cu_2_MnSnS_4_ nanocrystals	240 min	85%	—	Solvothermal method	74
Cu_2_SnS_3_/RGO nanocomposites	140 min	92%	—	Solvothermal route	65
ZnO/Cu_2_SnS_3_ nanorod array film	90 min	90%	3.87 × 10^−2^ min^−1^	Controllable one-step electrodeposition process	75
Cu_2_SnS_3_ + GO composite	240 min	88%	1.13 × 10^−3^ min^−1^	Precipitation technique	76
Cu_2_SnS_3_ nanoparticles	120 min	95%	0.0021 min^−1^	Hydrothermal method	15
Cu_2_SnS_3_ thin film	180 min	90%	0.012 min^−1^	Ultrasonic spray pyrolysis	77
rGO–Cu_2_SnS_3_ composites	60 min	94.1%	9.0 × 10^−2^ min^−1^	Facile *ex situ* process	58
Cu_2_SnS_3_ nanoparticles	120 min	90%	—	One-pot thermal decomposition method	78
Cu_2_SnS_3_/Ti^3+^–TiO_2_ p–n heterojunction	90 min	98%	—	Hydrothermal method	79
Flower-like Cu_2_SnS_3_/RGO hybrid material	210 min	87%	—	Hydrothermal route	66
Cu_2_SnS_3_ nanostructures	180 min	92%	0.0135 min^−1^	Template-free hydrothermal process	14
***Gd-doped CTS thin film***	***220 min***	***90.77%***	***0.093 min*** ^***−1***^	***Spin coating***	***This work***

## 4. Conclusions

This study investigated Cu_2_Sn_1−*x*_Gd_*x*_S_3_ (CTGS) thin films synthesized *via* spin coating, focusing on how varying the Gd/Sn ratio affects their structural, optical, and photocatalytic properties. Gd doping led to notable structural changes, including suppressing the cubic (112) phase and the appearance of prominent CuS peaks. Crystallite sizes for the (112) orientation were 53.37 nm (CTS) and 51.51 nm (CTGS-2), while those for the (106) orientation ranged from 43.30 to 50.26 nm. These shifts are attributed to increased lattice distortion and crystallite defects due to Gd incorporation. Raman spectroscopy showed slight peak shifts, likely caused by stoichiometric fluctuations from Sn and S volatilization. Morphological analysis revealed that Gd altered the films' dense, flake-like grain structure, affecting compactness and surface uniformity. Optical measurements showed 20–27% transmittance values in UV-Vis for CTS, CTGS-1, and CTGS-2, with a marked decrease around 664 nm. The CTGS-3 film showed very low transmittance (∼1%) at lower wavelengths, rising to ∼24% in the infrared. Band gap values (2.00–2.10 eV for most films, and 1.50 eV for CTGS-3) suggest the formation of secondary phases such as SnS_2_ and CuS. Photocatalytic tests using methylene blue under alkaline conditions (pH 10) showed degradation efficiencies of 86.16%, 84.37%, 80.76%, and 90.77% for 0%, 1%, 2%, and 3% Gd doping, respectively. The highest-performing catalyst (3% Gd) also showed strong activity across pH 4–10. Scavenger experiments further confirmed the involvement of reactive species in the degradation process. Overall, Gd doping significantly influences CTS thin films' structural, optical, and photocatalytic properties, highlighting their promise for photodegradation applications in wastewater treatment.

## Conflicts of interest

The authors declare that they have no known competing financial interests or personal relationships that could have appeared to influence the work reported in this paper.

## Data Availability

The data supporting this study's findings are available from the corresponding author upon reasonable request.

## References

[cit1] Keller J. (2024). *et al.*, High-concentration silver alloying and steep back-contact gallium grading enabling copper indium gallium selenide solar cell with 23.6% efficiency. Nat. Energy.

[cit2] Deepa K., Ramamurthy P. C., Singha M. K. (2019). Mesoporous Cu2ZnSnS4 nanoparticle film as a flexible and reusable visible light photocatalyst. Opt. Mater..

[cit3] Kişnişci Z. (2024). *et al.*, Electrical properties of Al/CZTSe nanocrystal Schottky diode. J. Mater. Sci.: Mater. Electron..

[cit4] Rahman S. and Das H., Performance Analysis of CZTS & CZTSe Based Thin Film Solar Cell by Changing Materials Property, MS thesis, University of Dhaka, Dhaka, Bangladesh, 2022

[cit5] Kişnişci Z. (2024). *et al.*, Structural and optical properties of Cu2ZnSnSe4 nanocrystals thin film. Opt. Quantum Electron..

[cit6] Jeganath K. (2022). *et al.*, Probing the depth inhomogeneity of spray pyrolyzed CZTS thin films via chemical etching. Inorg. Chem. Commun..

[cit7] Paris M. (2014). *et al.*, Solid-state NMR and Raman spectroscopy to address the local structure of defects and the tricky issue of the Cu/Zn disorder in Cu-poor, Zn-rich CZTS materials. Inorg. Chem..

[cit8] Shin D., Saparov B., Mitzi D. B. (2017). Defect engineering in multinary earth-abundant chalcogenide photovoltaic materials. Adv. Energy Mater..

[cit9] Jili N., Dlamini N., Mola G. T. (2023). Computational investigation of the effect ZnS buffer layer on the hole transport of polymer solar cell. Phys. B.

[cit10] Dakua P. K. (2024). *et al.*, Evaluating CZTS solar cell performance Based on generation and recombination models for possible ETLs through numerical analysis. J. Electron. Mater..

[cit11] Keerthana S. (2024). *et al.*, Synthesis of surfactant assisted Cu2ZnSnS4 (CZTS) photocatalysts for removal of dyes from wastewater. Sustain. Energy Technol. Assessments.

[cit12] Zhong J. (2015). *et al.*, Biomolecule-assisted solvothermal synthesis of 3D hierarchical Cu2FeSnS4 microspheres with enhanced photocatalytic activity. Appl. Surf. Sci..

[cit13] Machale A. R. (2021). *et al.*, Facile hydrothermal synthesis of Cu2SnS3 nanoparticles for photocatalytic dye degredation of mythelene blue. Mater. Today: Proc..

[cit14] Zaman M. B., Poolla R. (2020). Morphological tuning of hydrothermally derived visible light active Cu2SnS3 nanostructures and their applications in photocatalytic degradation of reactive industrial dyes. Opt. Mater..

[cit15] Shelke H. D. (2022). *et al.*, Multifunctional Cu2SnS3 nanoparticles with enhanced photocatalytic dye degradation and antibacterial activity. Materials.

[cit16] Ayub A. (2025). *et al.*, Advancing Dye Degradation: Integrating Microbial Metabolism, Photocatalysis, and Nanotechnology for Eco-Friendly Solutions. Bacteria.

[cit17] Chen S. (2008). *et al.*, Preparation, characterization and activity evaluation of p–n junction photocatalyst p-ZnO/n-TiO2. Appl. Surf. Sci..

[cit18] Naknonhan S. (2025). *et al.*, Pivotal role of CaCO3 in Ca/ZnO photocatalyst for promoting the degradation of trichlorophenol. J. Environ. Chem. Eng..

[cit19] Seremak W. (2025). *et al.*, Durability assessment of low-pressure cold-sprayed TiO2 photocatalytic coatings: Photocatalytic and mechanical stability. Surf. Coat. Technol..

[cit20] Li C. Q., Wang J. J. (2024). Copper sulfide based photocatalysts, electrocatalysts and photoelectrocatalysts: innovations in structural modulation and application. Small.

[cit21] Zu B. (2024). *et al.*, Engineering of Cu-Based Quaternary Sulfide Nanomaterials for Photocatalytic Applications. Photocatal.: Res. Potential.

[cit22] Umehara M. (2013). *et al.*, Cu2Sn1-xGexS3 (x= 0.17) thin-film solar cells with high conversion efficiency of 6.0%. Appl. Phys. Express.

[cit23] Dursun S., Akyıldız H., Kalem V. (2023). Production of CuCoO2 nanoparticle/SnO2 nanofiber heterostructures for visible light photocatalytic applications. J. Photochem. Photobiol., A.

[cit24] Dursun S. (2023). *et al.*, Investigation of photocatalytic activity (under visible light) of ultrathin CZTS films produced in different thicknesses by PLD method. Opt. Quantum Electron..

[cit25] Chang S.-K. (2024). *et al.*, Rapid pH-dependent photocatalytic degradation of methylene blue by CdS nanorods synthesized through hydrothermal process. Arabian J. Chem..

[cit26] Hanafi M. F., Sapawe N. (2020). Effect of pH on the photocatalytic degradation of remazol brilliant blue dye using zirconia catalyst. Mater. Today: Proc..

[cit27] Jiang Y. (2017). *et al.*, Effect of Cd content and sulfurization on structures and properties of Cd doped Cu2SnS3 thin films. J. Alloys Compd..

[cit28] Hayashi H. (2020). *et al.*, Influence of Ge/(Ge+ Sn) composition ratio in Cu2Sn1-xGexS3 thin-film solar cells on their physical properties and photovoltaic performances. Sol. Energy Mater. Sol. Cells.

[cit29] Maeta H. (2025). *et al.*, Deposition of single-phase monoclinic Cu2SnS3 thin films on Mo-coated substrates by dual-source fine-channel mist CVD. Jpn. J. Appl. Phys..

[cit30] Wan J. (2025). *et al.*, Harnessing Halogen-Induced Anharmonic Effect to Achieve Low Lattice Thermal Conductivity in High-Symmetry Cu2SnS3 for High-Performance Thermoelectric Applications. Adv. Funct. Mater..

[cit31] Baturay Ş. (2025). *et al.*, Influence of Gd Doping on Cu2Sn1-xGdxS3 Thin Film Solar Cell. iScience.

[cit32] Magdy W. (2023). *et al.*, Correlation between some physical properties of pure and Sb doped Cu2SnS3 thin films under the effect of sulfur amount for solar cell application. Mater. Chem. Phys..

[cit33] Patterson A. (1939). The Scherrer formula for X-ray particle size determination. Phys. Rev..

[cit34] Banotra A., Padha N. (2017). Effect of annealing on physical characteristics of the vacuum evaporated mixed phase SnxSy thin films. Mater. Res. Express.

[cit35] Dolma P. (2022). *et al.*, Sequentially evaporated layer deposition stack of CuxS thin films for photonics applications. J. Mater. Res. Technol..

[cit36] Suryawanshi P. (2021). *et al.*, A simple chemical approach for the deposition of Cu2SnS3 (CTS) thin films. Mater. Today: Proc..

[cit37] Bhise S. M. (2024). *et al.*, Sulfur-annealed Cu2SnS3 (CTS) thin films for solar cell applications. J. Mater. Sci.: Mater. Electron..

[cit38] Pallavolu M. R. (2019). *et al.*, Effect of sulfurization time on the performance of monoclinic Cu2SnS3 solar cells. Sol. Energy.

[cit39] Gadha M. K. (2025). *et al.*, Annealing temperature optimization for dip-coated Cu2SnS3 thin films: Sustainable pathway to CTS/Zn (O, S) solar cells via numerical simulation. Mater. Sci. Eng., B.

[cit40] Parkin I., Rowley A. (1993). Metathesis routes to tin and lead chalcogenides. Polyhedron.

[cit41] Chen D. (2004). *et al.*, Microwave-assisted polyol synthesis of nanoscale SnSx (x= 1, 2) flakes. J. Cryst. Growth.

[cit42] Heidariramsheh M. (2021). *et al.*, Optoelectrical and structural characterization of Cu2SnS3 thin films grown via spray pyrolysis using stable molecular ink. Sol. Energy.

[cit43] Fernandes P., Salomé P., Da Cunha A. (2010). A study of ternary Cu2SnS3 and Cu3SnS4 thin films prepared by sulfurizing stacked metal precursors. J. Phys. D: Appl. Phys..

[cit44] Amorim C. (2024). *et al.*, Cu3BiS3 film synthesis through rapid thermal processing sulfurization of electron beam evaporated precursors. Emergent Mater..

[cit45] Che H. (2021). *et al.*, Metallization of polymers by cold spraying with low melting point powders. Surf. Coat. Technol..

[cit46] Zhang B. (2020). *et al.*, Inverse vulcanization below the melting point of sulfur. Mater. Chem. Front..

[cit47] Hossain E. S. (2019). *et al.*, Fabrication of Cu2SnS3 thin film solar cells by sulphurization of sequentially sputtered Sn/CuSn metallic stacked precursors. Sol. Energy.

[cit48] Sayed M. H., Gomaa M. M., Boshta M. (2023). Effect of Ge doping on the material properties of sprayed Cu2SnS3 thin films. Results in Optics.

[cit49] Chalapathi U., Poornaprakash B., Park S.-H. (2019). Antimony induced crystal growth for large-grained Cu2SnS3 thin films for photovoltaics. J. Power Sources.

[cit50] Miyata Y., Nakamura S., Akaki Y. (2015). Effects of H2S annealing on Cu-Sn-S thin films prepared from vacuum-evaporated Cu-Sn precursor. Phys. Status Solidi C.

[cit51] Becerra R. (2014). *et al.*, One-step diffusion membrane assisted CBD synthesis and characterization of Cu2SnS3 thin films. J. Phys.: Conf. Ser..

[cit52] Laghchim E. (2025). *et al.*, Investigating the impact of copper precursors on the photovoltaic performance of Cu2SnS3 thin film-based solar cells toward an enhanced power conversion efficiency of 9.85%. Sol. Energy.

[cit53] Boudouma A. (2023). *et al.*, A one-step electrodeposition method was used to produce monoclinic Cu2SnS3 thin films for the development of solar cells. J. Mater. Sci.: Mater. Electron..

[cit54] Patel B. (2018). *et al.*, Electrical properties modulation in spray pyrolysed Cu2SnS3 thin films through variation of copper precursor concentration for photovoltaic application. J. Anal. Appl. Pyrolysis.

[cit55] Chaudhari J., Joshi U. (2018). Fabrication of high quality Cu2SnS3 thin film solar cell with 1.12% power conversion efficiency obtain by low cost environment friendly sol-gel technique. Mater. Res. Express.

[cit56] Shelke H. D. (2020). *et al.*, Influence of deposition temperature on the structural, morphological, optical and photoelectrochemical properties of CBD deposited Cu2SnS3 thin films. J. Alloys Compd..

[cit57] Zaman M. B. (2023). *et al.*, Non hydrazine based chemical synthesis of earth abundant Cu2SnS3 thin film photocatalyst for wastewater treatment. Ceram. Int..

[cit58] Olatunde O. C., Onwudiwe D. C. (2022). Synthesis of reduced graphene oxide/copper tin sulfide (Cu2SnS3) composite for the photocatalytic degradation of tetracycline. J. Inorg. Organomet. Polym. Mater..

[cit59] Shelke H. (2022). *et al.*, Multifunctional Cu2SnS3 nanoparticles with enhanced photocatalytic dye degradation and antibacterial activity. Materials.

[cit60] Maheskumar V., Vidhya B. (2018). Investigation on the morphology and photocatalytic activity of Cu3SnS4 synthesized by ball milling and solvothermal method. J. Photochem. Photobiol., A.

[cit61] Toloman D. (2017). *et al.*, Impact of Gd ions from the lattice of TiO2 nanoparticles on the formation of reactive oxygen species during the degradation of RhB under visible light irradiation. Mater. Sci. Semicond. Process..

[cit62] Li H. (2018). *et al.*, Fabrication of bismuth molybdate photocatalyst co-substituted by gadolinium and tungsten for bismuth and molybdenum: design and radical regulating by the synergistic effect of redox centers and oxygen vacancies for boosting photocatalytic activity. J. Taiwan Inst. Chem. Eng..

[cit63] Shanthi R. V. (2022). *et al.*, Optical, structural and photocatalytic properties of rare earth element Gd3+ doped MgO nanocrystals. Chem. Phys. Lett..

[cit64] Olatunde O. C., Onwudiwe D. C. (2022). Selective syntheses of kuramite (Cu2SnS3) and petrukite (Cu3SnS4) phases of copper tin sulphide, and their electrochemical and photocatalytic properties. Results Mater..

[cit65] Vadivel S. (2016). *et al.*, Biomolecule-assisted solvothermal synthesis of Cu 2 SnS 3 flowers/RGO nanocomposites and their visible-light-driven photocatalytic activities. RSC Adv..

[cit66] Yao S. (2017). *et al.*, Enhanced photocatalytic degradation of Rhodamine B by reduced graphene oxides wrapped-Cu2SnS3 flower-like architectures. J. Alloys Compd..

[cit67] Madkour M. (2024). *et al.*, Surface and electrochemical characteristics of S-scheme nanoheterostructured photocatalysts of AgIO3/Cu2SnS3 with enhanced solar energy driven photocatalytic activity. Surf. Interfaces.

[cit68] Machale A. R. (2022). *et al.*, Influence of hydrothermal temperature on the structural, morphological, optical and photocatalytic properties of ternary Cu2SnS3 nanoparticles. Chem. Phys. Lett..

[cit69] Yang Q. (2023). *et al.*, Enhanced activation of H2O2 by bimetallic Cu2SnS3: A new insight for Cu (II)/Cu (I) redox cycle promotion. J. Colloid Interface Sci..

[cit70] Rahaman S. (2020). *et al.*, Effect of copper concentration on CTS thin films for solar cell absorber layer and photocatalysis applications. Superlattices Microstruct..

[cit71] Meenakshisundaram S. P., Sridharan M. B. (2024). Effect of capping ligands and reaction temperature on the structural, morphological, and photocatalytic properties of Cu2SnS3 nanostructures. J. Alloys Compd..

[cit72] Xu K. (2024). *et al.*, Construction of ZnS/Cu2SnS3 heterojunction for photocatalytic degradation of organic pollutant methylene blue. Mater. Lett..

[cit73] Zhang Z. (2024). *et al.*, Preparation of Z-type Cu2SnS3/g-C3N4 heterojunction material and its synergistic photocatalytic performance with H2O2. J. Environ. Chem. Eng..

[cit74] Guan H. (2017). *et al.*, Photocatalytic and thermoelectric properties of Cu2MnSnS4 nanoparticles synthesized via solvothermal method. Mater. Lett..

[cit75] Guo Y. (2016). *et al.*, Construction of ZnO/Cu 2 SnS 3 nanorod array films for enhanced photoelectrochemical and photocatalytic activity. RSC Adv..

[cit76] Shelke H. D. (2023). *et al.*, Enhanced photocatalytic activity of the Cu2SnS3+ GO composite for the degradation of navy blue ME2RL industrial dye. Coatings.

[cit77] Rahaman S., Singha M. K. (2022). Nanoarchitectonics earth-abundant chalcogenide Cu 2 SnS 3 thin film using ultrasonic spray pyrolysis for visible light-driven photocatalysis. Appl. Phys. A:Mater. Sci. Process..

[cit78] Ben Smida Y. (2022). *et al.*, Synthesis of Cu9S5, SnS2, and Cu2SnS3 Nanoparticles from Precursor Complexes and Their Photodegradation Activities on Methyl Orange. J. Inorg. Organomet. Polym. Mater..

[cit79] Shahzad K. (2021). *et al.*, Synthesis of novel pn heterojunction Cu2SnS3/Ti3+-TiO2 for the complete tetracycline degradation in few minutes and photocatalytic activity under simulated solar irradiation. Ceram. Int..

